# Reproductive Seasonality in *Nesticus* (Araneae: Nesticidae) Cave Spiders

**DOI:** 10.1371/journal.pone.0156751

**Published:** 2016-06-09

**Authors:** Linnea M. Carver, Patricia Perlaky, Alan Cressler, Kirk S. Zigler

**Affiliations:** 1 Department of Biology, Sewanee: The University of the South, Sewanee, Tennessee, United States of America; 2 Raccoon Mountain Caverns, Chattanooga, Tennessee, United States of America; 3 Independent Researcher, Atlanta, Georgia, United States of America; United States Department of Agriculture, Beltsville Agricultural Research Center, UNITED STATES

## Abstract

Spiders of the family Nesticidae are members of cave communities around the world with cave-obligate (troglobiotic) species known from North America, Europe, Asia and the Indo-Pacific. A radiation of *Nesticus* (Araneae: Nesticidae) in the southern Appalachians includes ten troglobiotic species. Many of these species are of conservation interest due to their small ranges, with four species being single-cave endemics. Despite conservation concerns and their important role as predators in cave communities, we know little about reproduction and feeding in this group. We addressed this knowledge gap by examining populations of two species on a monthly basis for one year. We made further observations on several other species and populations, totaling 671 individual spider observations. This more than doubled the reported observations of reproduction and feeding in troglobiotic *Nesticus*. Female *Nesticus* carry egg sacs, facilitating the determination of the timing and frequency of reproduction. We found that *Nesticus* exhibit reproductive seasonality. Females carried egg sacs from May through October, with a peak in frequency in June. These spiders were rarely observed with prey; only 3.3% (22/671) of individuals were observed with prey items. The frequency at which prey items were observed did not vary by season. Common prey items were flies, beetles and millipedes. Troglobiotic species constituted approximately half of all prey items observed. This result represents a greater proportion of troglobiotic prey than has been reported for various troglophilic spiders. Although our findings shed light on the life history of troglobiotic *Nesticus* and on their role in cave ecosystems, further work is necessary to support effective conservation planning for many of these rare species.

## Introduction

Spiders of the family Nesticidae are members of cave communities around the world with cave-obligate (troglobiotic) species known from North America, Europe, Asia and the Indo-Pacific [1–6]. Troglobiotic nesticids have reduced eyes and pigmentation relative to surface species [2]. Two radiations of troglobiotic nesticids are known in the United States, one of *Nesticus* (Araneae: Nesticidae) in the southern Appalachians and another of *Eidmanella* (Araneae: Nesticidae) in Texas [2,7–8].

The southwestern Appalachians are a hotspot for cave biodiversity with high levels of troglobiotic species richness and endemism [9–10]. *Nesticus* spiders are a significant component of this diversity. The radiation of *Nesticus* in the southeastern United States comprises around thirty described species including ten troglobionts from Tennessee, Alabama and Georgia. Four species are single-cave endemics, and, given their extremely limited ranges, are of significant conservation interest [2,8]. Many other members of this radiation are troglophiles (or ‘eutroglophiles’ after Culver and Pipan [11]). Troglophiles are facultative cave inhabitants; they may complete their entire life cycle underground but can also be found in similar habitats outside of caves [12]. Previous work on these spiders focused on phylogenetics [13–14] and population genetics [15].

**Fig 1:**
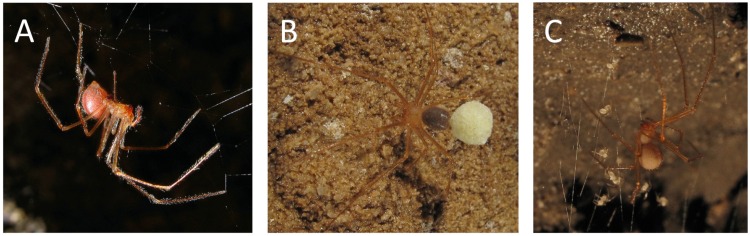
Photos of troglobiotic *Nesticus*. (A) *N*. *barri* female in web (The Marlow Holes, Franklin County, Tennessee); (B) *N*. *stygius* with egg sac (Obe Lee Cave, Overton County, Tennessee); and (C) *N*. *furtivus* with spiderlings (Raccoon Mountain Caverns, Hamilton County, Tennessee). All photos by Alan Cressler.

Despite the important role of troglobiotic arachnids as predators in cave ecosystems, little is known of the ecology and life history of *Nesticus* and other troglobiotic arachnids [16]. Most ecological and behavioral observations of troglobiotic *Nesticus* are anecdotal. *Nesticus* form tangle webs on the walls and ceilings of caves, from which they hang in an inverted position and wait for prey (Fig 1A). Webs are often constructed along ceilings of stream corridors, in small concavities in rock walls, and in crevices where a mud bank meets the cave wall. A dozen observations of prey items have been reported including troglobiotic millipedes, springtails and beetles as well as troglophilic flies and juvenile cave crickets [17–18].

After laying eggs, females carry them in an egg sac attached to the spinnerettes at the back of the abdomen (Fig 1B). Observations of females carrying egg sacs have been reported in the literature suggesting a trend toward reproduction in the late summer [8,17–18]. With the exception of Mays’ [18] monthly observations of *N*. *barrowsi*, these observations were not acquired in a systematic fashion. Egg number is reported to vary from 20 to 58 in troglobiotic *Nesticus* of the Appalachians [8,17–18]. Once the spiderlings leave the egg sac they can be observed in the mother’s web (Fig 1C).

We addressed knowledge gaps in the biology of troglobiotic *Nesticus*. This was motivated by their important ecological role as predators in cave communities and the significant conservation interest in the group. We made monthly observations of reproduction and feeding in two troglobiotic *Nesticus* species–*N*. *barri* and *N*. *furtivus*–for a year. *N*. *barri* is known from caves across four counties in Tennessee and Alabama and *N*. *furtivus* is a single-cave endemic from Tennessee. We made further observations of several other poorly known species and populations in Tennessee, Alabama and Georgia. These observations greatly expand the available data on reproduction and feeding in troglobiotic *Nesticus* from the Appalachians. Our findings shed light on the role of *Nesticus* in cave ecosystems and on the life history of these spiders.

## Materials and Methods

### Scientific permits

Work in Tennessee was permitted by the Tennessee Wildlife Resources Agency (permit #1605). Work in Georgia was permitted by the Georgia Department of Natural Resources (permit #8934). Work in Horseskull Cave was permitted by the Southeastern Cave Conservancy.

### Field sites and species studied

**Fig 2:**
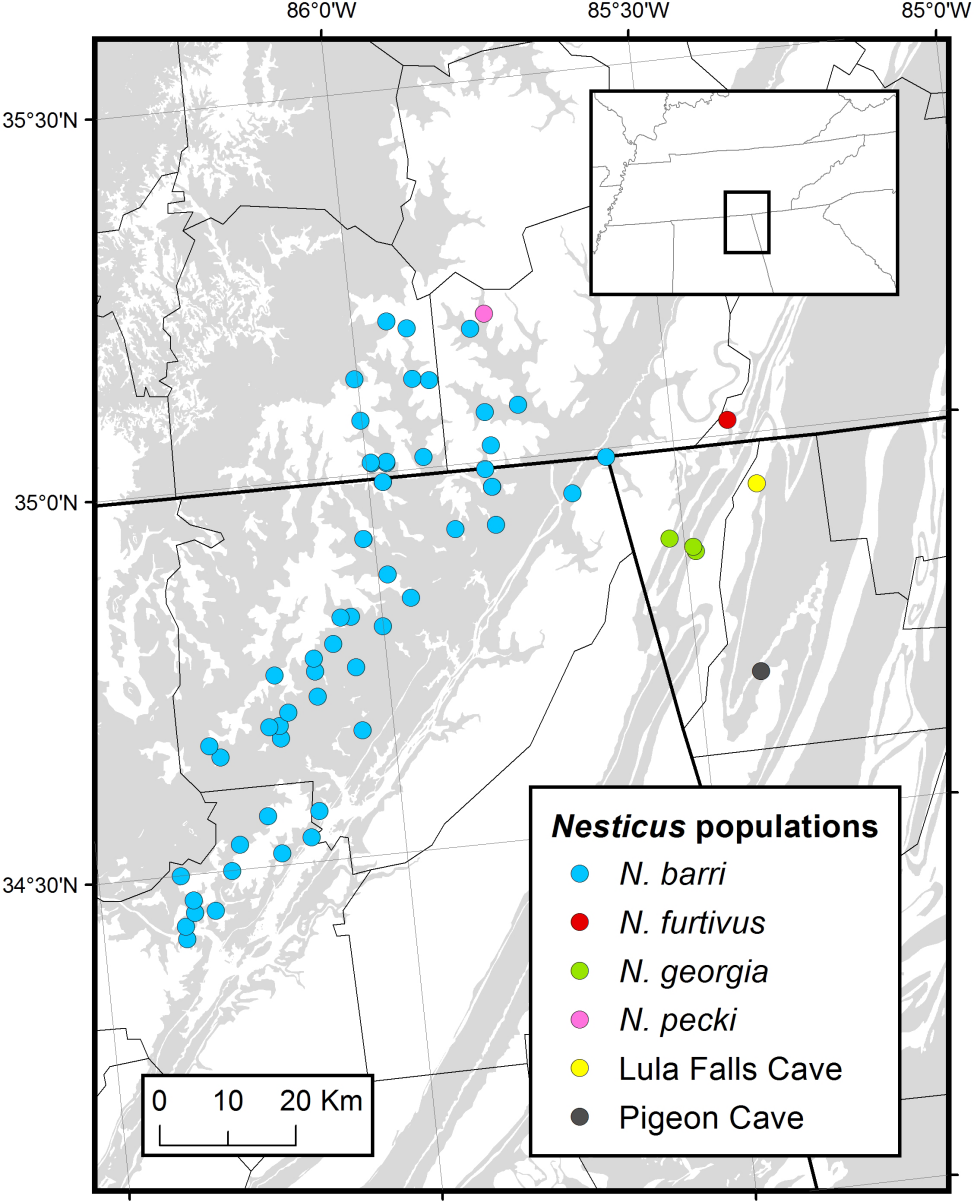
Troglobiotic *Nesticus* populations in the vicinity of the Tennessee, Alabama and Georgia junction. State and county boundaries are outlined. Gray background indicates karst topography, derived from Weary and Doctor [19].

We investigated reproductive seasonality and feeding in *Nesticus barri* and *N*. *furtivus* on a monthly basis for one year. *Nesticus barri* is known from more than 50 caves in Tennessee and Alabama (Fig 2). *Nesticus barri* was surveyed in Buckets of Blood Cave (Tennessee Cave Survey (TCS) FR61) in Franklin County, Tennessee. *Nesticus furtivus* was surveyed in Raccoon Mountain Caverns (TCS HM4) in Hamilton County, Tennessee. This is the type and only known locality for *N*. *furtivus* [2,8] (Fig 2). Additional surveys of other *Nesticus* species and populations were conducted one to three times in Horseskull Cave in Jackson County, Alabama (Alabama Cave Survey AJK613; *N*. *barri*), Monteagle Saltpeter Cave in Marion County, Tennessee (TCS MN24, the type and only known locality for *N*. *pecki*) [8], Sittons Cave in Dade County, Georgia (Georgia Speleological Survey (GSS) GDD9, *N*. *georgia*), Pigeon Cave in Walker County, Georgia (GSS GWK57, home to an undetermined species of *Nesticus*) and Lula Falls Cave in Walker County, Georgia (GSS GWK617, also home to an undetermined species of *Nesticus*). As a single species of troglobiotic *Nesticus* is known from each of these caves we were able to identify *Nesticus* species by locality.

### Data collection

We limited our investigation to the transition and deep zones of caves [20]. We searched for spiders using headlamps on all accessible cave surfaces including walls, floor, ceiling and breakdown. The sex and maturity of each spider was noted. Mature males had distinctively enlarged and sclerotized pedipalps. Mature females had a prominent and protruding epigynum. Immature males had enlarged pedipalps that were pale and unsclerotized. Immature females had an incompletely developed and non-protruding epigynum. For spiders that had not yet developed sex-specific characteristics, or in cases where they could not be confidently determined, sex and maturity were recorded as ‘undetermined’. In most cases the sex and maturity of the spiders could be determined without disturbing the spider. In cases where the spider’s position made this difficult, spiders were briefly captured in a shell vial and examined with a 10x magnification hand lens before being released at the point of collection. We also noted whether or not the spider was in a web. The presence of egg sacs or spiderlings was recorded, as was the presence of prey items. All observations were made by one of the authors and all authors had extensive experience observing cave spiders. We did not attempt to estimate population sizes in our surveys; instead we aimed to determine the frequency of reproduction and feeding in these populations throughout the year. The results of each cave trip are presented in Table 1. Egg sacs were collected from *N*. *barri* in Buckets of Blood and Horseskull caves and dissected to count eggs. We lost the data from the December 18, 2013 visit to Raccoon Mountain Caverns.

**Table 1:** All *Nesticus* observations from this study. State, county and cave survey numbers for caves are presented in the methods.

Species	Locality	Date	Total observed	Sex and Maturity					Egg sacs	Prey items
				Male		Female		Undetermined		
				Mature	Immature	Mature	Immature			
*Nesticus barri*	Buckets of Blood Cave	30-Jan-13	14	1	1	8	1	3	0	0
	Buckets of Blood Cave	13-Feb-13	37	5	3	20	1	8	0	1
	Buckets of Blood Cave	27-Mar-13	24	6	0	12	0	6	0	0
	Buckets of Blood Cave	24-Apr-13	40	4	4	15	0	17	0	1
	Buckets of Blood Cave	25-May-13	24	2	1	11	0	10	1	1
	Buckets of Blood Cave	26-Jun-13	26	0	0	15	0	11	9	1
	Buckets of Blood Cave	17-Jul-13	31	3	4	13	1	10	6	0
	Buckets of Blood Cave	30-Aug-13	56	6	7	24	2	17	7	2
	Buckets of Blood Cave	25-Sep-13	50	2	11	10	5	22	2	0
	Buckets of Blood Cave	31-Oct-13	35	6	10	10	2	7	0	2
	Buckets of Blood Cave	18-Nov-13	42	8	4	15	3	12	0	0
	Buckets of Blood Cave	24-Dec-13	20	7	1	6	0	6	0	1
	Buckets of Blood Cave	27-Jan-14	24	6	5	8	2	3	0	0
	Horseskull Cave	20-Feb-13	9	0	0	5	0	4	0	0
	Horseskull Cave	14-Jul-13	34	1	2	19	1	11	9	2
	Horseskull Cave	17-Sep-13	22	0	2	14	0	6	7	1
*N*. *furtivus*	Raccoon Mountain Caverns	24-Feb-13	3	0	1	1	0	1	0	1
	Raccoon Mountain Caverns	31-Mar-13	5	1	1	3	0	0	0	0
	Raccoon Mountain Caverns	28-Apr-13	17	0	6	6	0	5	0	2
	Raccoon Mountain Caverns	24-May-13	13	0	2	6	0	5	0	0
	Raccoon Mountain Caverns	28-Jun-13	9	2	1	4	0	2	1	0
	Raccoon Mountain Caverns	18-Jul-13	15	1	4	5	0	5	1	3
	Raccoon Mountain Caverns	29-Aug-13	2	1	0	1	0	0	0	0
	Raccoon Mountain Caverns	20-Sep-13	14	2	3	6	1	2	1	3
	Raccoon Mountain Caverns	30-Oct-13	8	0	0	1	0	7	1	0
	Raccoon Mountain Caverns	17-Nov-13	36	0	1	7	0	28	0	1
	Raccoon Mountain Caverns	18-Dec-13	No data	No data	No data	No data	No data	No data	No data	No data
	Raccoon Mountain Caverns	28-Jan-13	4	1	0	1	0	2	0	0
*N*. *georgia*	Sittons Cave	28-Apr-13	2	0	0	2	0	0	0	0
*N*. *pecki*	Monteagle Saltpeter Cave	1-Sep-13	6	0	0	2	0	4	1	0
*N*. sp.	Lula Falls Cave	8-Aug-13	35	4	8	8	6	9	0	0
*N*. sp.	Pigeon Cave	8-Aug-13	14	0	0	9	2	3	8	0

### Other sources of data

When possible we incorporated previously published information into our analyses. Observations of troglobiotic *Nesticus* with egg sacs are reported from several previous studies [8,17–18]. Only two studies detailed the number of mature females, mature males and immatures. Mays [18] used the same methods as this study in surveying a troglobiotic *Nesticus* species on a monthly basis for a year, totaling 430 observations of *N*. *barrowsi*. Mays [18] also recorded prey items. Hedin and Dellinger [8] conducted four surveys totaling 56 observations of *N*. *furtivus*. These surveys were done in different years, once in April and July and twice in August. Observations of *N*. *barri*, *N*. *furtivus* and *N*. *barrowsi* taken on a monthly basis for a year constituted 85% of all spider observations considered, limiting any effect of seasonality on the results. The number of eggs per egg sac has been reported for several troglobiotic *Nesticus* species from the southern Appalachians [8,17–18]. Observer bias in these studies is limited as the information collected about each spider (sex, presence/absence of egg sac, presence/absence of prey) is straightforward.

## Results

### General observations

We made 32 cave visits between January 2013 and January 2014 resulting in 671 *Nesticus* observations (Table 1). The majority of our observations were of *N*. *barri* (*N* = 488 spiders observed) and *N*. *furtivus* (*N* = 126). Additional observations were made of several other *Nesticus* species and populations (*N* = 57). Across all populations we observed mature females (*N* = 267) nearly four times as often as mature males (*N* = 69).

### Reproduction

**Fig 3:**
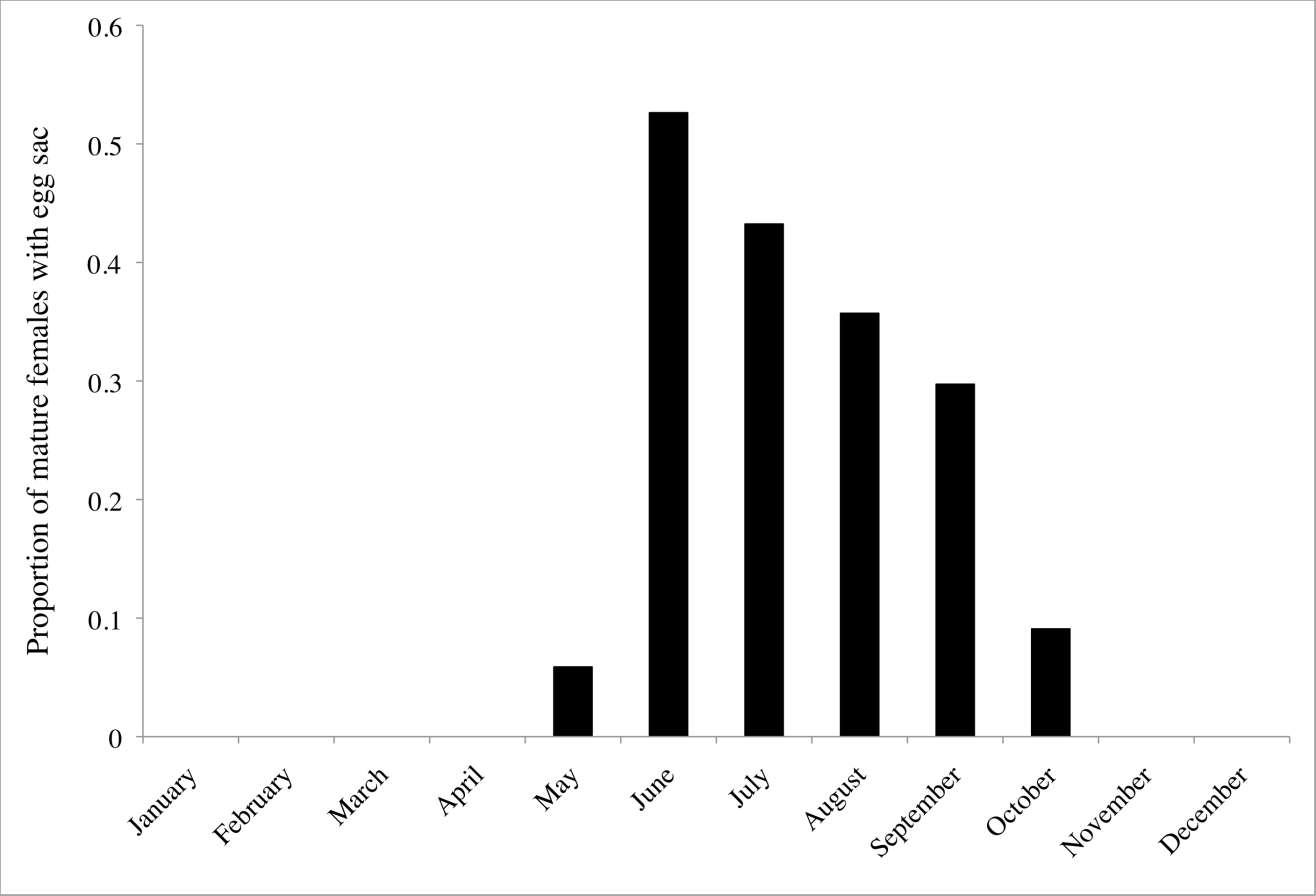
Proportion of mature *Nesticus* females observed with egg sacs each month. Data collected in this study for *N*. *barri*, *N*. *furtivus*, *N*. *georgia*, *N*. *pecki* and *Nesticus* sp. from Lula Falls Cave and Pigeon Cave.

**Fig 4:**
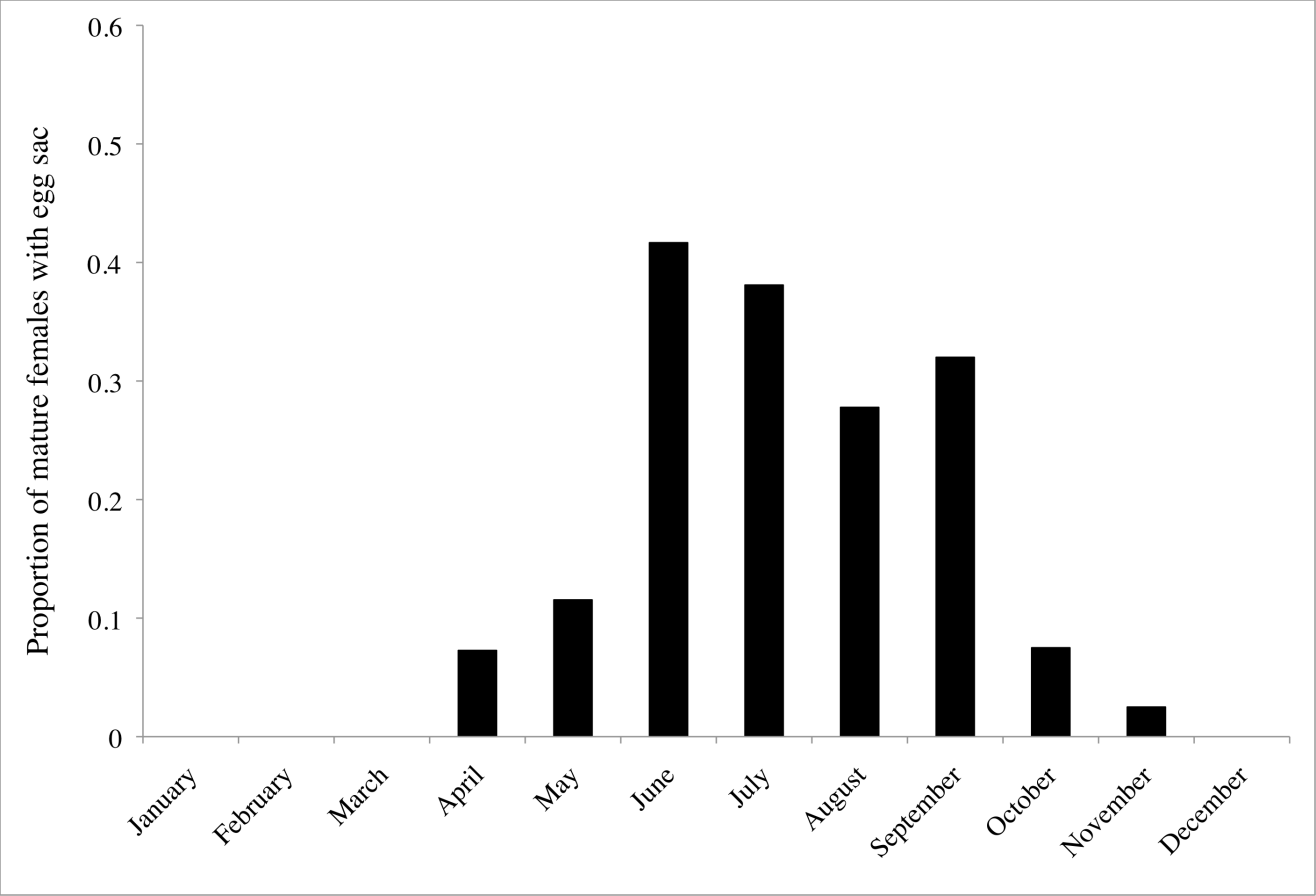
Proportion of mature *Nesticus* females observed with egg sacs each month. Data combined from this study and previous studies [8,18].

Across all the observed populations, 20.2% (54/267) of mature females were observed with egg sacs. Troglobiotic *Nesticus* exhibited reproductive seasonality in the species and populations studied. We observed egg sacs from May through October. The frequency of egg sacs peaked in June, when more than 50% of mature females carried egg sacs (Fig 3). When we combined our data with previous observations from the literature [8,18] a similar pattern of reproductive periodicity was present with egg sacs first observed in April, peaking in frequency in June, and continuing to be present at low frequency into November (Fig 4). On three occasions we observed mature male and female *Nesticus* in the same web. We observed a pair of *N*. *barri* mating on April 24, 2013 in Buckets of Blood Cave. We observed 26 spiderlings in a *N*. *furtivus* web on November 17, 2013 and an undetermined number of spiderlings in a *N*. *barri* web on July 13, 2013.

Reproduction in three troglobiotic *Nesticus* species has been surveyed in a single cave on a monthly basis for a year–*N*. *barri* (this study), *N*. *furtivus* (this study) and *N*. *barrowsi* [18]. Of those species, mature females were observed with egg sacs at a higher frequency in *N*. *barri* (15.0%, 25/167) than in either *N*. *furtivus* (9.8%, 4/41) or *N*. *barrowsi* (10.1%, 19/188), but this difference was not significant (*X*^*2*^ (2, *N* = 396) = 2.2, *p* = 0.33).

The mean number of eggs in *N*. *barri* egg sacs collected during this study was 39, with a range of 22 to 66 (*N* = 7; Table 2). The mean number of eggs per egg sac previously reported in the literature for five troglobiotic *Nesticus* species from the southern Appalachians was 38, with a range from 20 to 58 (*N* = 13; Table 2) [8,17–18].

**Table 2:** Number of eggs per egg sac for troglobiotic *Nesticus* from the southern Appalachians.

Species	Locality	Eggs	Source
*Nesticus barri*	Buckets of Blood Cave	22, 29, 36, 37, 40, 66	This study
	Horseskull Cave	43	This study
	Moody Cave	35	[17]
*N*. *barrowsi*	Gregorys Cave	28, 32, 34, 37, 48	[18]
*N*. *dilutus*	Grassy Creek Cave	20, 41	[8]
*N*. *georgia*	Sittons Cave	41, 44, 54, 58	[17]
*N*. *stygius*	Raven Bluff Cave	22	[8]

### Feeding

**Fig 5:**
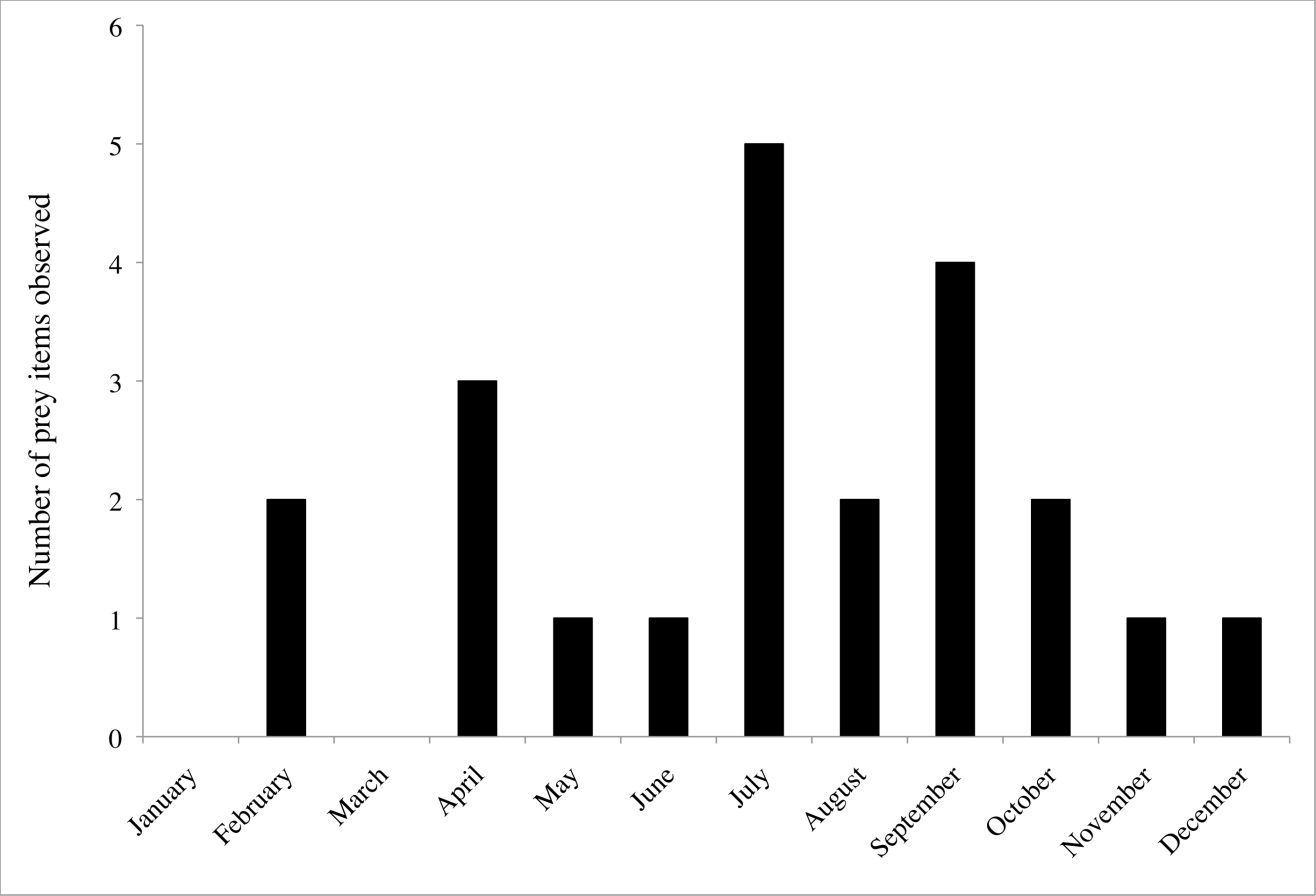
Number of prey items observed monthly for all *Nesticus* populations and species observed.

3.3% (22/671) of spiders were observed with prey. At least one spider with prey was observed each month with the exception of January and March (Fig 5). Troglobiotic *Nesticus* fed on flies (Heleomyzidae and other families), beetles (*Ptomaphagus hatchi* and other unidentified species) and millipedes (*Pseudotremia* sp. and *Scoterpes* sp.) (Table 3). Dipterans represented 50% (11/22) of prey items observed. When the two populations were compared, *N*. *furtivus* was observed with prey at a higher frequency (8.7%, 11/126) than *N*. *barri* (1.9%, 8/423) (*X*^*2*^ (1, *N* = 549) = 11.62, *p* = 0.0007).

**Table 3:** Prey observations for troglobiotic *Nesticus* from the southern Appalachians.

Species	Locality	Prey	Source
*Nesticus barri*	Buckets of Blood Cave	*Pseudotremia minos* (Diplopoda, Cleidogonidae) (*N* = 2), *Ptomaphagus hatchi* (Coleoptera, Leiodidae) (*N* = 1), Heleomyzidae (Diptera) (*N* = 4), unknown (*N* = 1)	This study
	Horseskull Cave	*Scoterpes stewartpecki* (Diplopoda, Trichopetalidae) (*N* = 1), unidentified beetle (*N* = 2)	This study
*N*. *barrowsi*	Gregorys Cave	*Scoterpes blountensis* (Diplopoda, Trichopetalidae) (*N* = 6), dipterans (*N* = 2), unknown (*N* = 1)	[18]
*N*. *furtivus*	Raccoon Mountain Cave	*Pseudotremia* sp. (*N* = 2), Heleomyzidae (Diptera) (*N* = 5), other dipterans (*N* = 2), unidentified beetle (*N* = 1), unknown (*N* = 1)	This study
*N*. *georgia*	Sittons Cave	*Ptomaphagus whiteselli* (Coleoptera, Leiodidae), *Pseudosinella hirsuta* (Collembola, Entomobryidae), Gryllacrididae (Orthoptera)	[17]

Although more prey items were present in the summer and fall (June through November) than in the winter and spring (December to May) (Fig 5), the frequency that we observed prey items did not differ between summer-fall and winter-spring (*X*^*2*^ (1, *N* = 671) = 0.01, *p* = 0.92). During the summer and fall, 3.4% (15/435) of spiders had prey items. In the winter and spring, 3.0% (7/236) of spiders had prey items.

## Discussion

### Reproduction

We observed reproductive seasonality with troglobiotic *Nesticus* reproducing in the summer and fall (Fig 3). This was consistent with previous studies that reported the number of egg sacs and mature females (Fig 4; [8,18]). This was also consistent with published and unpublished observations noting the presence of egg sacs, all of which were observed from May to November ([8,17]; P. Perlaky and A. Cressler unpublished observations). Although reproductive seasonality was evident, we may have overestimated the frequency of females with egg sacs. The white egg sacs are conspicuous (Fig 1B) and this may lead to a sampling bias due to increased detection of females with egg sacs relative to individuals without egg sacs.

Our observations of a mating pair of *N*. *barri* in April, *N*. *barri* spiderlings in a web in July and *N*. *furtivus* spiderlings in a web in November are also consistent with a reproductive cycle running through the summer and fall. Other reports of *N*. *furtivus* spiderlings are from July, August and September (P. Perlaky and A. Cressler unpublished observations). Our observations suggest females carry egg sacs for four to six weeks. Similarly, Ives [21] reported that females of troglophilic *N*. *carteri* carried egg sacs for slightly more than a month until spiderlings emerged. Across five troglobiotic *Nesticus* species, there was a range of 20 to 66 eggs per egg sac (Table 2) with a mean of 38 eggs per egg sac.

From the data collected in this study and by Mays [18] we can estimate how often troglobiotic *Nesticus* reproduce. With 12.1% (48/396) of mature females observed with egg sacs in monthly surveys, females carrying egg sacs for four to six weeks, and a sampling bias favoring the detection of females with egg sacs, it appears that mature females produce around one egg sac per year. As we did not track individual spiders it is possible that some mature females produce no egg sacs in a year whereas others produce more than one egg sac in a year. With an average of 38 eggs per egg sac (Table 2), we estimate a mature female produces ~40 eggs per year.

The physical environment and food supply in temperate caves vary seasonally due to changes in surface temperature and precipitation [12,22]. This seasonal variation is thought to influence reproduction in troglobionts. Several examples of reproductive seasonality in troglobionts from the eastern United States are known. Kane et al. [23] found larvae and pupae of a predatory cave beetle from the Mammoth Cave system were most common in the early spring. They ascribed this pattern to seasonal variation in food availability to adult beetles (in the form of cricket eggs). Similarly, a troglobiotic crayfish and several species of cave fish from the eastern United States maintain annual reproductive cycles and release young in the summer, when food availability may be greatest [24–26]. However, not all troglobionts from the eastern United States exhibit reproductive seasonality. Year-round reproduction has been reported for several species of round fungus beetles (Coleoptera: Leodidae) [27] and aquatic isopod species show no consistent pattern of annual reproductive seasonality (summarized in [28]).

Work conducted by Ives [21,29] on *N*. *carteri* presents an important contrast to the reproductive seasonality we observed in troglobiotic *Nesticus*. Ives [21] monitored reproduction in a population of *N*. *carteri* in Three Springs Cave (Hamblen County, Tennessee) on a monthly basis for six years. *N*. *carteri* ranges from Indiana to Virginia. It is troglophilic and can be found in cave and surface habitats (Gertsch 1984). Ives [21] found that *N*. *carteri* in Three Springs Cave reproduced year-round. The proportion of spiders with an egg sac varied from 19% to 38% per month. Assuming some of the spiders he observed were male and/or immature, the proportion of mature females carrying an egg sac would have been higher. Thus, reproduction in this population of troglophilic *N*. *carteri* differed from what we observed in troglobiotic *Nesticus* species, as they reproduced more frequently and lacked reproductive seasonality. Unfortunately, Three Springs Cave was flooded after the construction of a hydroelectric dam in 1942, precluding further study of this population.

### Feeding

Despite their important role as predators in cave ecosystems, little is known about the diet of troglobiotic spiders, including *Nesticus*. Previous observations of *N*. *barrowsi* and *N*. *georgia* identified a range of prey items including millipedes, flies, beetles, springtails and juvenile cave crickets [17–18]. Consistent with those reports, we observed millipedes (*Scoterpes* and *Pseudotremia*), flies (family Heleomyzidae and others) and beetles (*Ptomaphagus* and others) in webs of *N*. *barri* and *N*. *furtivus* (Table 3). While food availability has been suggested to drive reproductive seasonality in some troglobionts, we did not find support for that pattern, as the frequency of prey in webs did not differ by season. However, the small number of prey items observed (22 total) limited our ability to detect seasonal differences.

The diet of troglobiotic *Nesticus* differed significantly from that of the troglophilic spiders *Meta ovalis*, *M*. *menardi*, *M*. *bourneti* and *Metellina merianae*. In caves, the troglophilic spiders fed largely on trogloxenic and troglophilic prey [30–32], only rarely capturing troglobiotic prey [33]. In contrast, approximately half of the observed prey of troglobiotic *Nesticus* was troglobiotic (beetles, millipedes and springtails). The other half of the observed prey was troglophilic flies and crickets (Table 3). Similarly, Mays [18] observed that *N*. *barrowsi* also fed predominantly on troglobiotic prey (six of nine observed prey items were troglobiotic millipedes, Table 3). Troglobiotic *Nesticus* are thus more deeply integrated into cave-specific food webs than troglophilic spiders. This observation highlights how cave food webs may differ with distance from a cave entrance. Troglobiotic spiders (and troglobiotic prey) are typically encountered deeper in a cave, whereas troglophilic spiders (and troglophilic prey) are more likely to be encountered close to an entrance.

### Diversity and Endemism

The *Nesticus* radiation in the southeastern United States includes some of the rarest spiders in North America. Of the ten troglobiotic species described from Tennessee, Alabama and Georgia, four are single-cave endemics. Several other species are known from fewer than five caves. We surveyed two undescribed populations which may represent new species and other undescribed populations are known. Short-range endemic invertebrate species such as these troglobiotic *Nesticus* are typically of great conservation interest [34–35].

In the course of this study we observed two single-cave endemic *Nesticus* species. We made repeated observations of *N*. *furtivus* at Raccoon Mountain Caverns, a commercial cave in Hamilton County, Tennessee. Of the single-cave endemic *Nesticus* species it is undoubtedly the best known. Although known only from this cave, and never observed in large numbers, the cave’s large size (> 8 km of passage, much of which is rarely visited) and the careful attention of the cave manager (P. Perlaky) and the cave owner confer a significant degree of protection to the species. We made a single observation of *N*. *pecki*, a single-cave endemic from Marion County, Tennessee. In contrast to *N*. *furtivus*, *N*. *pecki* is extremely poorly known. Our observation of *N*. *pecki* in September 2013 was, to our knowledge, the first observation of the species in more than twenty years [8]. We observed six spiders, including one female carrying an egg sac, in the vicinity of the small second entrance. Similar to our observations, Hedin and Dellinger [8] reported seeing fewer than ten spiders on visits in 1991 and 1992, suggesting *N*. *pecki* is rare even within its only known locality. As suggested by Hedin and Dellinger [8], further study of caves and similar habitats nearby could clarify whether the range of *N*. *pecki* extends beyond this cave.

We observed two *Nesticus* populations that may represent new species. *Nesticus* populations were reported from two caves on Pigeon Mountain in Walker County, Georgia [36]. We visited Pigeon Cave in August 2013 and observed 14 spiders, including eight females with egg sacs. With no other *Nesticus* known from Pigeon Mountain, it is likely these eyeless spiders represent an undescribed species. The second unidentified *Nesticus* population was discovered by one of the authors (A. Cressler) in Lula Falls Cave on Lookout Mountain in Walker County, Georgia. We observed 35 individuals during a visit in August 2013. Although located less than 15 km from populations of *N*. *georgia* and *N*. *furtivus*, the Lula Falls Cave population is clearly distinct, as they have eyes whereas *N*. *georgia* and *N*. *furtivus* are eyeless. Searches of several caves in the immediate vicinity of Lula Falls Cave have not identified other *Nesticus* populations. As with the Pigeon Mountain populations, we suspect the Lula Falls Cave population represents an undescribed *Nesticus* species. Specimens from both populations were collected and shared with spider systematists to facilitate the determination and/or description of these species.

Like most short-range endemic species, troglobiotic *Nesticus* have limited dispersal abilities and are confined to discontinuous habitats. This has resulted in remarkable diversification within *Nesticus* of the southern Appalachian region with six species from Tennessee, Alabama and Georgia known from five or fewer caves. Little is know about most of these species. This is exemplified in this study by *N*. *pecki*, which to our knowledge has been observed only once since it was described [8]. Basic information on population sizes, prey items, reproduction and habitat threats is lacking for most of these species. For species known from more than one cave, we lack information about connectivity between cave populations (see [15] for an exception). Surveys of caves in the vicinity of known populations might uncover new populations of known species. All of this information would inform conservation assessments of these species. As indicated by this study, all of these projects are feasible as the spiders are reasonably conspicuous members of cave communities.

### Conclusion

With ten described troglobiotic species and numerous troglophilic species, *Nesticus* spiders are part of many cave communities in the southern Appalachians. While surface and troglophilic *Nesticus* species generally have large ranges, many troglobiotic *Nesticus* are short-range endemics, with numerous species known from one or a few caves; several cave populations of *Nesticus* that likely represent undescribed species are also known. We found that troglobiotic *Nesticus* exhibit reproductive seasonality, reproducing during the summer and fall with mature females producing an average of one egg sac per year. Troglobiotic *Nesticus* feed on beetles, millipedes, flies and other invertebrates. In contrast to cave-inhabiting troglophilic spiders which rarely capture troglobiotic prey, around half of observed prey items were troglobiotic, likely a result of troglobiotic *Nesticus* being part of food webs deeper in caves than troglophilic spiders. Despite this contribution to our understanding of the ecology of cave spiders, further study of *Nesticus*, in particular on the short-range endemic members of the genus, is critical to ensuring the proper management of these rare species.
